# Role of trained immunity in DCs and macrophages in the induction of Th2 responses and allergy treatment. What do we know?

**DOI:** 10.3389/fimmu.2026.1748337

**Published:** 2026-02-05

**Authors:** Hannah Ruth Schiller, Carola Zeigermann, Stefan Schülke

**Affiliations:** 1 Allergology Research Section, Allergology Division, Paul-Ehrlich-Institut, Langen, Germany; 2 Central Animal Unit, Veterinary Division, Paul-Ehrlich-Institut, Langen, Germany

**Keywords:** allergen-specific immunotherapy, allergy, APC, dendritic cell, epigenetics, macrophage, metabolism, Th2 response

## Abstract

In a process termed trained immunity activated dendritic cells (DCs) and macrophages undergo distinct metabolic changes that contribute to their effector function: While certain activated DC subsets and M1 macrophages undergo a switch towards higher rates of glycolysis and a “disrupted Krebs cycle” to produce important immune effector molecules, alternatively activated (M2) macrophages, plasmacytoid DCs (pDCs), and conventional DCs type 1 (cDC1s) can rely on oxidative phosphorylation for their effector function. DCs and macrophages are also important cells in allergic reactions. While the induction of trained immune responses by microbial stimuli and vaccines is meanwhile well characterized, the contribution of trained immunity to either the establishment, elicitation, or treatment of allergic responses is largely unknown. In this context, recent results suggest distinct trained immunity responses to be established in allergic children. Here it seems that infections early in life predispose to the latter development of allergies, and trained immunity to also contribute to the immune modulation occurring in allergic patients during allergen-specific immunotherapy. Therefore, better understanding of trained immunity in these antigen-presenting cell (APC) subsets may allow to establish new biomarkers and enable a more targeted and efficient treatment of allergic diseases. This article summarizes the specific immune metabolic alterations observed in activated DCs and macrophages explaining their connection to DC and macrophage effector function. It then discusses our current knowledge on the contribution of trained immune responses in the establishment and treatment of allergic diseases.

## 1 Introduction

Allergic diseases continue to have a significant impact on the health and well-being of affected patients worldwide. Therefore, the interest in effective and safe therapeutic approaches remains high.

In allergic reactions, the role of the adaptive immune system has been long established. In 1963, it was categorized by Coombs and Gell into four different types based on the underlying pathomechanisms (1). Type I allergy is the most common form and represents an IgE-mediated hypersensitivity reaction to otherwise harmless environmental allergens. During allergic sensitization, naive cluster of differentiation (CD)4^+^ Th2 cells are activated by antigen-presenting dendritic cells (DCs), which in turn stimulate allergen-specific B cells to produce IgE. These allergen-specific IgE antibodies bind to mast cells, eosinophils, and basophils via the high-affinity IgE receptor Fc_ϵ_RI [reviewed in (2)]. Upon secondary exposure to the allergen, cross-linking of membrane-bound IgE molecules on these cells leads to cell degranulation and the release of inflammatory mediators [reviewed in (3)]. Clinically, this acute inflammatory reaction manifests within a few minutes, which is why it is also referred to as immediate-type allergy. Depending on the site of exposure, a wide variety of symptoms can occur, ranging from inconvenient local reactions to life-threatening anaphylactic reactions [reviewed in (4)]. In contrast, interaction of allergens via the low affinity IgE receptor Fc_ϵ_RII expressed on various hematopoietic cells such as B cells, monocytes, DCs, and epithelial cells results in enhanced endocytosis/phagocyotis and antigen-presentation while also being involved in the regulation of serum IgE levels [reviewed in (5)].

Recent studies suggest that the innate immune system may also influence the establishment and subsequent severity of allergic reactions. In this context, the concept of ‘trained immunity’ describes the ability of the innate immune system to develop memory-like responses through metabolic and epigenetic reprogramming of innate immune cells after a primary stimulation. This allows for a stronger immune response to be initiated upon secondary contact with either the same or a different stimulus [reviewed in (6)]. This increased responsiveness is observed both locally and systemically. At the local level, it is characterized by persistent priming of tissue-resident macrophages and other effector cells, which exhibit an enhanced inflammatory and antimicrobial response profile over a period of days to months. Systemically, long-term trained immunity is mediated by epigenetic reprogramming of hematopoietic stem- and progenitor cells in the bone marrow, resulting in the production of differentiated trained monocytes, macrophages, and NK cells exhibiting enhanced effector functions [reviewed in (7)]. In this way, a long-lasting, non-specific memory function of the innate immune system is established which likely contributes far more to the pathogenesis of type I allergies than has previously been assumed [reviewed in (8)].

Monocytes and DCs in particular can be trained by environmental factors such as microbial signals, pollutants, or dietary metabolites [reviewed in (9, 10)]. Such changes affect the expression of costimulatory molecules, cytokine production, and the presentation of allergen-derived peptides. A trained pro-inflammatory profile can support the differentiation of naive T cells towards a Th2 phenotype (11), thereby enhancing allergic responses. Conversely, certain microbial stimuli promote tolerogenic imprinting, which strengthens regulatory networks and reduces the risk of type I allergies (12).

In this review, we will briefly introduce the general trained immunity programs observed in DCs and macrophages, review the evidence of trained immunity contributing to the establishment of allergic diseases, and how it may contribute to the treatment of allergies.

## 2 General immune metabolic adaptations observed in DCs and macrophages

As observed for other immune cells [reviewed in (13, 14)], also DCs and macrophages undergo distinct metabolic changes upon activation that are essential in establishing trained immune responses. They can be broadly distinguished in (I) mainly glycolytic phenotypes observed in certain DC subsets and M1 macrophages characterized by high rates of glycolysis and a “disrupted Krebs cycle” and (II) e.g. the alternatively activated (M2) macrophages and conventional DCs type 1 (cDC1s) that mainly/also rely on glutamine- and fatty acid-driven oxidative phosphorylation (see below).

The main DC subsets in humans are conventional cDC1s, cDC2s, plasmacytoid DCs (pDCs), and monocyte-derived DC (moDCs). These cells and their molecular phenotype and functions have been extensively reviewed (15, 16). While their metabolic phenotypes in allergic diseases are currently understudied, recent studies have shed light on the different general metabolic adaptations in these cell types. A detailed description of the complex metabolic networks in the different DC subsets is not the focus of this paper and can be found in other recent reviews (15, 16), but we would like to give a brief overview about the function and metabolic adaptations in these cells.

cDC1s are specialized in cross-presenting antigens to CD8^+^ T cells via major histocompatibility complex (MHC)I while secreting the cytokines tumor necrosis factor (TNF)-α, interleukin (IL)-6, IL-12, as well as type I interferons to promote T cell activation [reviewed in (17–19)]. In contrast to other DC subtypes, cDC1s are characterized by the expression of “Toll”-like receptor (TLR)1, TLR3, TLR6, TLR8, and TLR10, giving them the capability to efficiently detect viral- and intracellular pathogens [reviewed in (20, 21)]. The main function of cDC2s is to present antigens to CD4^+^ T cells via MHCII molecules promoting their activation, the induction of type II and III immune responses, and the subsequent induction of CD8^+^ T cell responses [reviewed in (22)]. Furthermore, they are characterized by the secretion of IL-12 and IL-1β and can induce Th1-, Th12-, as well as Th17 responses (23, 24).

The currently available studies indicate cDC1s and cDC2s to be metabolically different. Compared to cDC2s, cDC1s seem to rely more strongly on oxidative phosphorylation than glycolysis. For example, TLR3- or TLR7/8 stimulation of cDC1s was found to result in increased mitochondrial content and oxidative phosphorylation (25) and splenic CD8α^+^ cDC1s displayed higher levels of higher oxidative phosphorylation (OXPHOS) than their CD8α^-^ (cDC2) counterparts (26). In contrast, cDC2s seem require ROS signaling for their differentiation (27) and higher levels of glycolysis and glutamine metabolism for their effector function [reviewed in (15, 16)]. However, further work is needed to provide a more detailed comparison of these DC subtypes along with their subpopulations (e.g. cDC2A and B).

pDCs specialize in anti-viral immune responses by producing large amounts of type I interferons [reviewed in (28)]. Interestingly, pDCs seem to be metabolically flexible as an RIG-I-like receptor (RLR)-induced and mammalian target of rapamycin (mTOR)-dependent increase in glutaminolysis driving oxidative phosphorylation was shown to be important for interferon I production, while TLR9-induced interferon I production was found to be dependent on glycolysis (29, 30).

moDCs differentiate from monocytes under inflammatory conditions and perform antigen presentation alongside the production of the pro-inflammatory cytokines TNF-α, IL-12, and IL-23 thereby promoting Th17 cell differentiation (31). However, they can also contribute to the initiation of Th2 responses and allergen-induced inflammation (32). In terms of metabolic phenotype, moDCs display high rates of iNOS- and mTOR-dependent glycolysis which was found crucial for their survival and effector function (30, 33). In contrast, fully mature moDCs show reduced protein synthesis, higher dependence on mitochondrial function, and lower glycolytic activity, demonstrating both the complexity and plasticity of metabolism in these cells (34).

Because of its relevance for allergic diseases (see part 3) we would like to focus on the mainly glycolytic phenotype observed in certain DC subsets and M1 macrophages. As sentinels of the immune system, DCs and macrophages have to identify and fight pathogens in infected tissues. These tissues are often characterized by a very low oxygen concentration. However, cellular mitochondrial metabolism relies on oxygen as terminal electron acceptor. Due to the unavailability of oxygen in acutely infected tissues, certain DC subsets (pDCs, moDCs, likely cDC2s, see above) and M1 macrophages switch their metabolism from mainly relying on oxidative phosphorylation while at rest, to the preferred generation of lactate from glucose when activated (35–38).

The generated lactate is subsequently exported from the cells, resulting in a pH reduction of the surrounding medium. This was termed the Warburg Effect after Otto Warburg who first observed this effect in cancer cells (39). While being energetically less efficient than completely metabolizing glucose in the Krebs Cycle, energy can be generated faster and independently of oxygen through lactate fermentation.

From experiments done on lipopolysaccharide (LPS)-stimulated macrophages we meanwhile know the switch towards Warburg metabolism to be mediated by four main events: (I) a TLR4-induced upregulation of the inducible nitric oxide (NO) synthetase (40). The generated NO molecules in turn nitrosylate the complexes of the mitochondrial electron transport chain, resulting in a shut-down of oxidative phosphorylation (41–43). (II) The TLR4-mediated activation of mTOR, a conserved seronine-threonine kinase that acts as a master regulator of both cellular metabolism and immune cell effector function (44). Among many other effects, mTOR triggers the activation of hypoxia-inducible factor 1 alpha (HIF-1α) which in turn promotes the transcription of glycolysis-related genes, resulting in enhanced rates of glycolysis (45, 46). (III) An upregulation of the glycolysis enzyme phospho-fructo kinase 2 that generates fructose-2,6-bisphosphat, acting as an allosteric activator of 6-phosphofructo-1-kinase, which further promotes glycolysis (47). (IV) Finally, TLR4 activation was reported to down-regulate AMP kinase which results in reduction of fatty acid oxidation (48).

**Figure 1:**
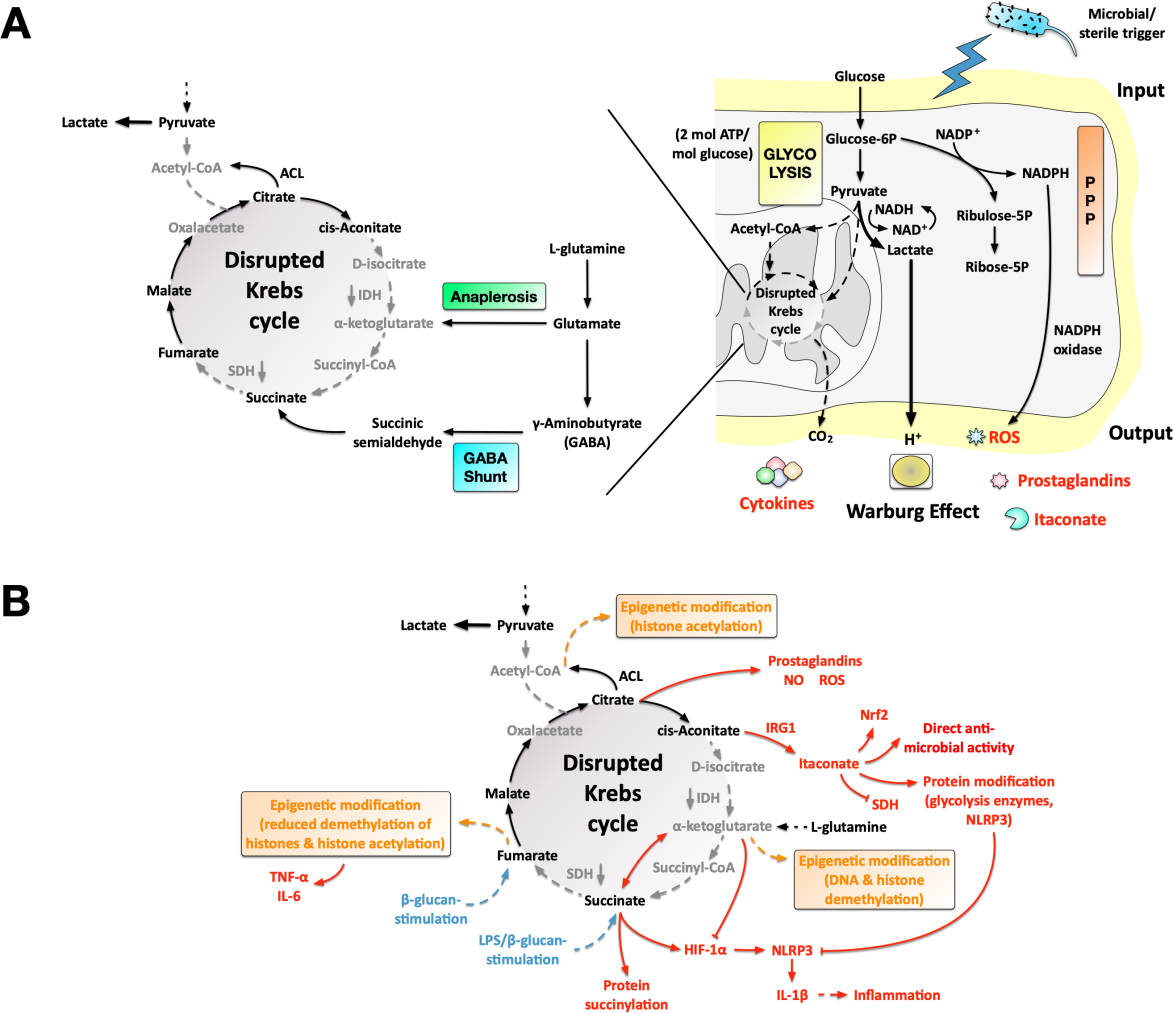
Principles of trained immune responses in DCs and macrophages displaying a mainly glycolytic phenotype. Certain dendritic cell (DC) subsets and macrophages undergo distinct metabolic changes upon activation which are essential for the establishment of immune responses. In infected tissues, DCs and macrophages need to fight pathogens under oxygen-deprived conditions that do not allow for efficient mitochondrial respiration. In monocyte-derived DCs, plasmacytoid DCs, conventional DCs type 2, and M1-macrophages, the glycolytic Warburg metabolism driven by recognition of either pathogens or sterile triggers results in a “disrupted Krebs cycle” due to the preferential generation of lactate from glycolysis-derived pyruvate **(A)**. Here, the accumulation of Krebs cycle intermediates (highlighted in black) in activated DCs and M1 macrophages is used to generate important immune effector molecules such as cytokines, reactive oxygen and -nitrogen species (ROS and NOS), prostaglandins, and the anti-microbial substance itaconate (shown in red in **(B)**). Besides being an anti-microbial substance itaconate has immune-modulatory effects via inhibition of succinate dehydrogenase (SDH, resulting in succinate accumulation), modifying either glycolysis enzymes or the inflammasome nucleotide-binding leucine-rich repeat receptor (NLR) family pyrin domain containing 3 protein (NLRP3), thereby reducing inflammasome-mediated inflammation, and activating the nuclear factor erythroid 2-related factor 2 (Nrf2). Additionally, accumulation of succinate acts as a danger signal resulting in the activation of hypoxia inducible factor 1α (HIF-1α) and downstream production of interleukin (IL)-1β. Also, succinate may be used as a substrate to post-translationally modify certain target proteins. α-ketoglutarate acts as a competitive agonist of succinate while also promoting epigenetic modifications. Finally, fumarate was shown to boost expression of tumor necrosis factor (TNF)-α and IL-6 by epigenetically modifying the respective promotors. Together with histone acetylation using citrate-derived acetyl-CoA, these processes result in the longer-term reprogramming of the cells via epigenetic changes, allowing these cells to mount more effective immune responses upon second contact with either the same or a different pathogen. In the pentose phosphate, NADPH oxidase-dependent ROS-production also contributes to pathogen clearance. For more information see main text. ACL, ATP citrate lyase; GABA, gamma amino butyric acid; IDH, iso-citrate dehydrogenase; IRG1, immune responsive gene 1; JAK, Janus kinase; NO, nitric oxide.

The thereby increased rates of glycolysis coupled with the generation of lactate result in a disrupted Krebs cycle” due to an undersupply of the mitochondrion with glycolysis-derived pyruvate (**Figure 1A**). In DCs and macrophages the disrupted Krebs cycle has been found to be locked at two distinct points: (I) at citrate due to a down-regulation of the isocitrate dehydrogenase and (II) at succinate due to a down-regulation of the succinate dehydrogenase (49) (**Figure 1A**). The disruption of the Krebs cycle at these point results in the accumulation of specific Krebs cycle intermediates. Fascinatingly, the accumulation of these molecules in certain activated APCs is elegantly used to generate important immune effector molecules such as cytokines, reactive oxygen and -nitrogen species (ROS and NOS), prostaglandins, and the anti-microbial substance itaconate. Here, acetyl-CoA generated from citrate is used for epigenetic modifications via acetylation of histones, citrate itself can be used to generate ROS, NOS, and prostaglandins, and cis-aconitate can be metabolized into itaconate, which besides being an anti-microbial substance has immune-modulatory effects by inhibiting the succinate dehydrogenase (resulting in succinate accumulation, see below), modifying either glycolysis enzymes or the inflammasome nucleotide-binding leucine-rich repeat receptor (NLR) family pyrin domain containing 3 protein (NLRP3), thereby reducing IL-1β, IL-18, and gasdermin D (GSDMD) maturation, and activating the transcription factor nuclear factor erythroid 2-related factor 2 (Nrf2) with its widespread down-stream signaling events [reviewed in (7, 50, 51)]. Additionally, accumulation of succinate was described to act as a danger signal, resulting in the stabilization of HIF-1α and downstream production of IL-1β (**Figure 1B**). Succinate may also be used as a substrate to post-translationally modify certain target proteins (52) [and reviewed in (53)]. Of note, α-ketoglutarate acts as a competitive agonist of succinate: it promotes the breakdown of HIF-1α via prolyl hydroxylase domain (PHD) enzymes, thereby reducing IL-1β maturation, and is a cofactor for both ten-eleven translocation (TET)-enzymes which can demethylate DNA, and Jumonji domain-containing protein-3 (JMJD3), an enzyme removing repressive DNA markers like histone 3 lysine 27 triple methylation (H3K27me3) (54). Finally, fumarate was shown to contribute to cell activation by inducing H3K4me3 at the promotors of the pro-inflammatory cytokines TNF-α and IL-6, as well as driving histone acetylation (H3K27 acetylation (H3K27ac)) (55) (**Figure 1B**).

Additionally, glycolysis-derived glucose-6P may be used in the pentose phosphate pathway: here especially NADPH oxidase-dependent ROS-production via the generated NADPH was shown to contribute to pathogen clearance (56) (**Figure 1B**). Whilst these events allow DCs and macrophages to effectively induce immune responses even in acutely inflamed, oxygen-deprived tissues, they also result in the longer-term cell reprogramming via epigenetic changes mediated by the above-described metabolic adaptations (e.g. acetyl-CoA being used for histone acetylation or fumarate used for the demethylation of TNF-α- and IL-6-promotors) [reviewed in (53)]. Together, these events leave a so-called “epigenetic scar”, referring to an altered chromatin structure in previously pathogen-experienced cells that allows these cells to mount more effective immune responses upon second contact with either the same or a different pathogen [reviewed in (7)].

**Figure 2:**
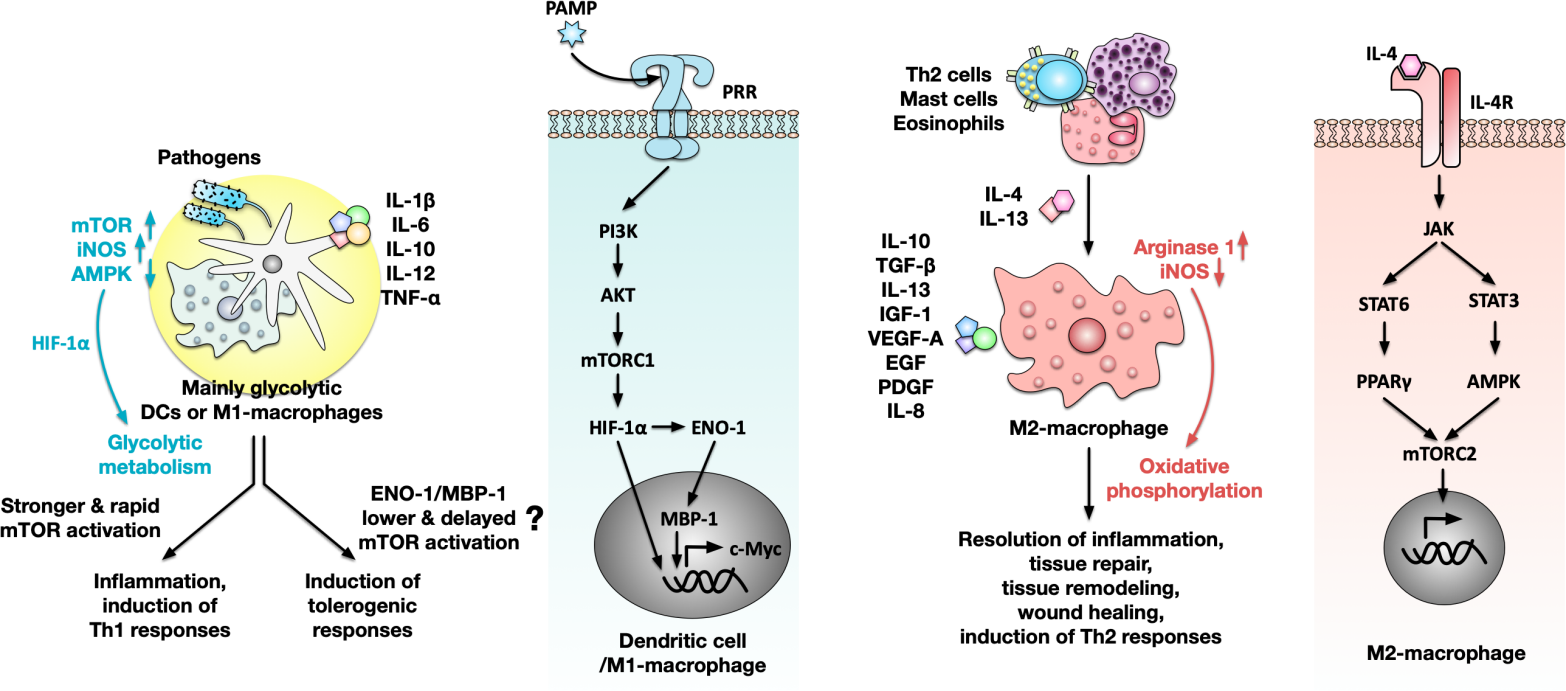
Immune metabolic regulation of effector function in different APC subsets. Certain DC subsets (monocyte-derived DCs, plasmacytoid DCs, likely conventional DCs type 2) and M1 macrophages react to pathogens/pathogen associated molecular patterns (PAMPs) via the activation of pattern recognition receptors (PRR) resulting in cell activation, secretion of pro-inflammatory cytokines, and induction of Th1-responses (left side). This PRR-induced cell differentiation is mediated via the phosphatidylinositol 3-kinase (PI3K)/protein kinase B (AKT)/mammalian target of rapamycin (mTOR)/hypoxia inducible factor (HIF)-1α/c-Myc oncogene (c-Myc)-axes. Antigen presenting cell (APC) activation can also result in a switch in cellular metabolism towards higher rates of glycolysis and the generation of lactate. This switch in cellular metabolic is mediated by an up-regulation of the inducible NO synthetase (iNOS) which results in a shut-down of oxidative phosphorylation, an activation of mTOR that promotes the HIF-1α-dependent up-regulation of glycolysis, and a down-regulation of the adenosine monophosphate kinase (AMPK) which results in reduction of fatty acid oxidation. Under certain conditions, activated APCs can also develop a tolerogenic phenotype. Here, possible switches may be enolase-1 (ENO-1)-mediated activation of c-Myc (which in turn promotes the formation of a tolerogenic phenotype) or the threshold and kinetics of mTOR signaling in these cells. In contrast, interleukin 4 receptor (IL-4R)-dependent M2-macrophages are responsible for resolution of inflammation, tissue repair, wound healing, tissue remodeling, and induction of Th2-responses via the secretion of the indicated cytokines (right side). This differentiation program is induced by signal transducer and activator of transcription (STAT)6 and STAT3, driving the mTOR complex 2 (mTORC2)-dependent activation of target genes. M2-macrophages express higher levels of arginase 1 and lower levels of iNOS than M1-macrophages promoting glutamine- and fatty acid-driven oxidative phosphorylation. For more information see text. EGF, epidermal growth factor; IGF-1, insulin-like growth factor 1; MBP-1, myc promoter-binding protein 1; PDGF, platelet-derived growth factor; PPAR, nuclear peroxisome proliferator activated receptor; TGF-β, transforming growth factor beta; VEGF-A, vascular endothelial growth factor A.

Currently, the metabolic mechanisms that contribute to the switch between inflammatory and tolerogenic APC phenotypes are not fully understood. Here, two candidate molecules were suggested: enolase-1 (ENO-1) and mTOR (**Figure 2**).

ENO-1 catalyzes the final step of glycolysis in the cell’s cytoplasm (57). Thereby, the activity of cytoplasmic ENO-1 enables rapid energy production to sustain inflammatory processes [reviewed in (58)]. Additionally, by using an alternative start codon, the ENO-1 gene can give rise to a truncated 37 kDa protein – myc promoter-binding protein 1 (MBP-1), that translocates into the nucleus where it binds to a master regulator of cellular growth, proliferation, and metabolism the c-Myc oncogene (c-Myc), thereby suppressing cell activation and proliferation and impeding for example tumor growth [(59, 60) and reviewed in (61)]. Since ENO-1 activity is also regulated by HIF-1α (62), activation of glucose metabolism in DCs and macrophages could therefore result in ENO-1/MBP-1-mediated induction of tolerogenic cells.

Furthermore, Wixler et al. reported the induction of tolerogenic mouse DCs (with the capacity to induce regulatory T cells) by stimuli like small spleen peptides, IL-10, or transforming growth factor beta (TGF-β) to be mediated by mTOR signaling (63). However, in this tolerogenic context mTOR activation was both delayed and weaker compared to classical LPS-mediated inflammatory mTOR activation, suggesting the difference in mTOR activation between immunogenic and tolerogenic stimuli to be rather quantitative than qualitative, depending on the activating threshold and kinetics of mTOR signaling pathway (63).

In contrast to the mainly glycolytic phenotype described above, a different metabolic phenotype is observed for M2-macrophages that are involved in resolution of inflammation, tissue repair and wound healing, but also tissue remodeling and induction of Th2 responses via the secretion of immune-suppressive cytokines like IL-10 and TGF-β, the Th2 cytokine IL-13, but also other cytokines and factors promoting angiogenesis [reviewed in (64)] (**Figure 2**). Mechanistically, M2-differentiation is driven by IL-4 receptor (IL-4R)-dependent activation of signal transducer and activator of transcription 6 (STAT6) and STAT3 driving mTOR complex 2 (mTORC2)-dependent activation of target genes, while M1-differentiation is promoted by pattern recognition receptor (PRR) activation and the phosphatidylinositol 3-kinase (PI3K)/protein kinase B (AKT)/mTOR/HIF-1α/c-Myc-axes [reviewed in (65)] (**Figure 2**). Consequently, M2-macrophages express higher levels of arginase 1 and lower levels of inducible NO synthetase (iNOS) than M1-macropages which results in these cells mainly relying on glutamine- and fatty acid-driven oxidative phosphorylation for energy generation [(66) and reviewed in (67)] (**Figure 2**).

Besides M2 macrophages, usage of oxidative phosphorylation has also been observed for the effector function of cDC1s (25, 26) and pDCs (29, 30).

## 3 Immune metabolic effects described in APCs in the context of allergies

Pathologically, type I allergies are driven by allergen-specific T- and B cells. However, our understanding of trained immune responses has shown, that the activation of these T- and B cells is also regulated by innate immune cells that undergo trained immune responses in response to certain triggers. This changes their long-term reactivity and thereby the T- and B cell responses induced. Therefore, it is not only possible but even likely that trained immunity also contributes to the establishment and maintenance of allergic responses. Consequently, better understanding these processes may also be result in optimized treatment of these diseases.

Recent studies have shown (I) allergic children to display specific changes in their innate immune responses that can be linked to trained immunity, (II) contact to certain pollutants and pathogens early in life to train innate immune cells, resulting in altered immune responses upon secondary contact with allergens, and (III) treatment of allergies by allergen-specific immunotherapy (AIT) to result in changes in the status of innate immune cells. These findings will be discussed in the following sections.

### 3.1 Differences in trained immunity status between allergic and non-allergic individuals

So far, multiple studies have shown changes in innate immune function (likely relying on trained immunity) early in life to promote the subsequent establishment of allergies (summarized in **Figure 2**). However, it has to be kept in mind, that these human studies so far are observatory in nature and did not apply a train-then-challenge design and provide only limited information on the induction of persistent epigenetic marks. Therefore, although indicative of the induction of trained immunity, further work is needed to conclusively demonstrate its involvement.

The immune system of the newborn is not fully functional and displays a Th2 bias caused by high levels of progesterone and Th2-promoting cytokines produced at the maternal-fetal interface [reviewed in (68)]. Th1 responses are only induced later in life upon contact with e.g. bacterial or viral pathogens [reviewed in (69)]. The formation of a healthy Th1-Th2 balance can be disturbed by e.g. interactions of the immune system with environmental factors such as either excessive hygiene, a dysfunctional (lung- and gut-) microbiome (for more information see the so-called “hygiene hypothesis”) or other factors like dysregulated myeloid-derived suppressor cells in the neonatal period [(70), reviewed in (69, 71)].

**Figure 3:**
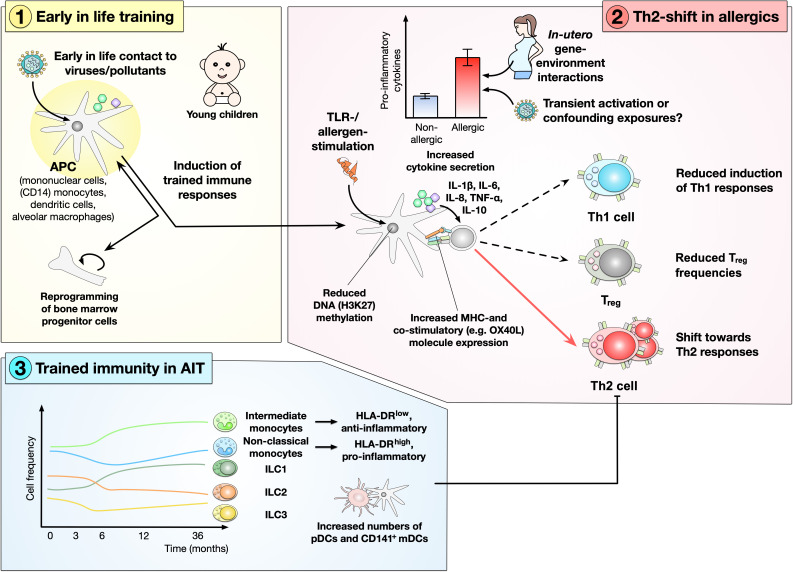
Relevance of trained immunity in the establishment and treatment of type I allergies. Early in life exposure to certain viruses and pollutants was shown to result in training of antigen-presenting cells (APCs) and likely also their bone-marrow progenitors. Upon secondary contact to either “Toll”-like receptor (TLR)-ligands or allergens this innate immune training results in enhanced inflammatory responses in allergic patients characterized by an enhanced production of pro- (e.g. interleukin (IL)-1β, IL-6, IL-8, tumor necrosis factor (TNF)-α) and anti-inflammatory (IL-10) cytokines and an increased expression of both MHC- and co-stimulatory molecules. Epigenetically, this APC hyperactivation was linked to a reduced methylation of histone 3 lysine 27 (H3K27). APCs activated under these conditions promoted Th2-responses while also reducing Th1- and Treg-responses. Trained immunity is also relevant in the treatment of allergies via allergen-specific immunotherapy (AIT). Here, AIT was shown to reduce the frequencies of innate-like lymphocytes type 2 (ILC2) while increasing the frequency of ILC1s. Also, the frequencies of anti-inflammatory intermediate monocytes were shown to increase alongside the numbers of plasmacytoid dendritic cells (pDCs) and CD141^+^ myeloid DCs (mDCs).

Already in 2011, Tulic and coworkers reported that (compared to healthy controls) allergic children react differently to TLR-activating stimuli in the first five years of life. Here, mononuclear cells from children that developed allergies within the first five years of life mounted exaggerated (IL-1β, TNF-α) cytokine responses at birth that declined with age, resulting in attenuated Th1 responses towards both allergens (house dust mite (HDM) and ovalbumin (OVA)) and mitogens (phytohemagglutinin (PHA)) at five years of age compared to age-matched healthy controls (72). This early immune system hyperresponsiveness was hypothesized to be caused by *in-utero* gene-environment interactions, resulting in a lack of Th1 maturation and predisposition towards allergic diseases (72). However, with the data currently available, it cannot be excluded that the increased inflammatory responses observed in allergies may also be caused by transient immune activation or other confounding exposures (**Figure 3**).

Moreover, infants who developed food allergy were shown to have increased monocyte to CD4^+^ T cell ratios with these monocytes also secreting higher amounts of pro-inflammatory cytokines (IL-1β, IL-6, TNF-α) after stimulation with LPS (73). These enhanced inflammatory responses were paralleled by lower frequencies of natural regulatory T cells, suggesting enhanced inflammatory responses early in life to shift T cells towards a Th2 phenotype while impairing Treg function (73). In line with this hypothesis, co-activation of isolated CD4 T cells with the mucosal cytokine TGF-β and IL-6 suppressed IL-2 secretion while at the same time promoting differentiation of T cells towards an IL-4-expressing, non-classical Th2 phenotype (73).

Also infants one year of age with a persistent egg allergy were shown to display changes in circulating (classical, non-classical, and intermediate) monocytes and DCs with both cell types displaying an increased activation status characterized by increased numbers, higher expression of HLA-DR, and higher production of pro- (IL-1β, IL-6, TNF-α, IL-8) and anti-inflammatory (IL-10) cytokines upon stimulation with LPS that continued into childhood (74). Interestingly, this was not the case in infants with transient egg allergy that also displayed higher serum vitamin D levels (74). These results were confirmed in a population-based cohort of challenge-confirmed, one year old egg allergic infants and aged-matched healthy controls, where purified CD14^+^ monocytes from egg allergic infants produced significantly higher amounts of IL-1β, IL-6, TNF-α, IL-10, GM-CSF, IL-8, and CC-chemokine ligand (CCL) 3 than the healthy controls (75). Also, South African children between one and three years of age with atopic dermatitis living in urban areas had elevated frequencies of eosinophils and monocytes with increased levels of IL-15, IL-22, Fms-related receptor tyrosine kinase 1 (Flt1), placental growth factor (PIGF), and basic fibroblast growth factor (βFGF) (76). They showed trained immune responses with a generalized pro-inflammatory status (76).

In peanut allergic patients secretion of IL-1β and IL-6 induced by both LPS and peanut protein was significantly increased from peripheral blood mononuclear cells (PBMCs) of food-allergic compared to non-allergic children five to ten years of age (77). This difference in reactivity was paralleled by a reduction in DNA methylation levels in allergic- compared to non-allergic patients (77), with a lower methylation status in genes associated with innate immune responses, balance between Th1- an Th2-responses, and seven genes not previously associated with food allergy (77). On the adaptive side of the immune system Martino et al. found differential methylation in children with food allergy in gene networks activated by the E2F family of transcription factors c-Myc, and mTOR, suggesting these changes in methylation level to skew T cell responses early in life (78).

Also, Lechner and colleagues described the enhanced production of pro-inflammatory mediators (IL-6, TNF-α, CCL17, leukotriene, prostaglandin E2 (PGE2)) from mouse macrophages isolated from both HDM-sensitized animals and asthmatic patients (79). In mice, HDM-induced allergic airway inflammation triggered a formyl peptide receptor 2- and TNF-dependent M2 macrophage phenotype with changes in metabolism (producing high amounts of 2-hydroxyglutarate, which promoted HIF-1α stabilization), histone demethylation, and production of the Th2-promoting chemokine CCL17 (79). These results obtained in mice suggested allergen-driven inflammation to induce TNF-dependent trained immunity which may exacerbate allergic airway inflammation. Also, HDM-allergic patients displayed reduced H3K27me3 levels (mediated by lysine demethylase 6B (KDM6B)) paralleled by an increased transcription of pro-inflammatory mediators in monocytes in response to low-dose LPS stimulation determined by transcriptomic analysis (80). These results further support the existence of trained immune responses in HDM-allergic children with a general pro-inflammatory bias.

Newborn children whose non-asthmatic mothers had low prenatal interferon (IFN)-γ:IL-13 ratios displayed differences in genome methylation especially in CpG sites enriched in microbe-responsive elements which was associated with increased frequencies of childhood asthma (81). In contrast to the studies detailed above, this was paralleled by an impaired PBMC responsiveness to LPS *in vitro* (characterized by reduced IL-6 secretion) and colonization with distinct, asthma-causing upper airway microbiota (81). Interestingly, these results link impaired anti-microbial responses in neonates to (I) altered epigenetics (and therefore the immune status of the mother), (II) trained immunity at birth, and (III) changes in microbiome later in life.

Recently, Chen et al. demonstrated that neonatal vaccination of mice with the Th2-promoting adjuvant aluminum hydroxide programmed the immune system towards subsequently mounting Th2 responses to a heterologous antigen while vaccination with the Th1-promoting adjuvant CpG resulted in preferential Th1 responses towards the same heterologous antigen (82). Mechanistically, these effects were shown to be caused by trained immunity and an altered ability of bone marrow DCs to prime Th2- and Th1-responses (82). Interestingly, neonatal application of the adjuvants but not application later in mouse life resulted in the Th2-preference (82), suggesting that the neonatal immune system is especially susceptible to initial immune training.

### 3.2 Infections with certain pathogens, bacterial vaccines, and contact to pollutants can modulate immune responses to allergens via training of DCs and macrophages

As discussed in the previous section, differences in innate immune system reactivity may predispose certain individuals to subsequent development of allergies later in life. In this chapter we discuss how immune responses to different infections, vaccines, and pollutants may either promote or prevent allergies.

Oral infection with enterovirus A71 (EV-A71) was found to correlate with increased risk of allergic disease in children in a study analyzing population-based national health insurance data from Taiwan (83). The incidence rate (per 1000 person-years) in the non-enterovirus-infected group *vs*. the enterovirus-infected group was 6.95 *vs*. 8.13 for atopic dermatitis, 32.18 *vs*. 41.78 for asthma, and 59.79 *vs*. 78.34, respectively for allergic rhinitis (83). In accordance with this observation, extrapulmonary EV-A71 infection in neonatal mice amplified HDM-induced airway inflammation (83). Mechanistically, bone marrow-derived macrophages (BMDMs) from animals previously infected with EV-A71 displayed a trained immune status characterized by increased secretion of IL-6 and TNF-α after challenge with HDM and a shift in cellular metabolism towards increased rates of glycolysis, that could be reversed by metabolic inhibitors such as 2-desoxy glucose (2-DG), metformin, or 5’-deoxy-5’-(methylthio) adenosine (MTA) (83). Also, *ex vivo*-isolated alveolar macrophages from EV-A71-trained mice challenged with HDM showed a trained M2 phenotype, that secreted higher amounts of IL-6 and TNF-α, and promoted Th2- and Th17-differentiation following allergen stimulation (83). Together these results suggest, that systemic trained immunity induced by EV-A71 infection in myeloid cells may promote asthma development later in life.

Epidemiological studies have also linked severe respiratory syncytial virus (RSV) infection with the development of persistent hyperreactive airway diseases, including asthma, later in life [(84) and reviewed in (85, 86)]. A study conducted by Sigurs et al. compared patients that were hospitalized with RSV bronchiolitis in infancy (RSV group) with control subjects. By the age of 13, 43% of the RSV group reported asthma/recurrent wheezing symptoms compared to only 8% of the control subjects. Furthermore, 39% of the RSV group *vs*. 15% of the control group reported allergic rhino conjunctivitis symptoms in the past 12 months. In the RSV group, sensitization to common inhaled allergens (determined by skin prick test or serum IgE antibodies) was more frequent (87). In this study, control subjects were recruited at the same age in the same winter season, and no demographic and environmental factors as well as difference in family history of atopy/asthma was found. In contrast to this, multiple studies did not find a significant difference between RSV hospitalized infants and control subjects by this age, which might be caused by differences in patient selection criteria (less severe diseases, later hospital admission (up to the age of 2)) or other factors such as climatic influences and allergen loads (88, 89).

Bone-marrow-derived DCs isolated from mice infected with RSV early in life displayed increased production of IL-6 as well as increased levels of the co-stimulatory molecules CD80, CD86, and the Th2-promoting OX40L compared to naive controls (90). In co-cultures with OVA-specific DO11.10 CD4^+^ T cells, these DCs induced thymic stromal lymphopoietin (TSLP)-dependent Th2- and Th17-responses (90). Therefore, these results suggest RSV infection to induce a dysregulated immune response that predisposes for allergic Th2-responses towards secondary antigens via TSLP-dependent epigenetic modifications, resulting in an enhanced risk of asthma development. Accordingly, in a mouse model of pneumonia virus of mice (PVM), a mouse equivalent strain of human RSV, viral co-infection with the allergen OVA was shown to result in metabolically reprogrammed trained macrophages that depended on proline biosynthesis and promoted non-Th2 (Th1/Th17) type allergic asthma following OVA challenge in neonatal mice (11).

In contrast, infection with the murid herpesvirus 4 (MuHV-4) prevented the development of HDM-induced experimental asthma in mice by triggering the replacement of virus-killed, resident lung alveolar macrophages with bone marrow-derived regulatory monocytes (producing IL-10 and expressing lower levels of co-stimulatory molecules) that repopulated the alveolar niche post-infection (91). This long-term training of the innate lung immune system blocked DCs from triggering HDM-induced Th2-responses one month after the initial infection (91).

While the effects of early viral infection on allergy development seem to depend on the specific virus (see above), early in life bacterial infection and application of bacteria-based vaccines seem to more generally protect from subsequent allergy development (92–96).

A population-based cohort study investigating 218,093 New South Wales-born Australian children suggested, that vaccination before four months of age with a whole-cell pertussis-containing vaccine, but not an acellular pertussis vaccine, decreased the risk of hospitalization due to food-induced anaphylaxis at ages 5 to 15 by 53% (92).

Similarly, Nieto et al. published a phase III randomized, double-blind, placebo-controlled, parallel-group trial sponsored by Inmunotek (Madrid, Spain) including a cohort of 120 children three years of age that reported >3 wheezing attacks during the previous year. Here, daily sublingual treatment over a period of six months with the bacterial vaccine MV130 significantly reduced symptom medication scores, the number of wheezing attacks, as well as their duration (93). MV130 is a heat-inactivated, whole-cell bacterial preparation that contains 90% gram-positive (*Streptococcus pneumoniae, Staphylococcus aureus, and Staphylococcus epidermidis*) and 10% gram-negative (*Klebsiella pneumoniae, Moraxella catarrhalis, and Haemophilus influenzae*) bacteria (93). In addition to T- and B cell mediated memory responses, MV130 induces heterologous protection via the induction of trained immune responses (97). In a follow-up mechanistic study Brandi and colleagues showed priming by MV130 to heterologously protect against both respiratory influenza A infection and systemic candidiasis also in T and B cell-deficient, Rag1-deficient mice, while also promoting enhanced cytokine production from mouse bone marrow progenitor cells and human monocytes, that relied on a shift in immune cell metabolism, all features typical for innate trained immune responses (98).

Also the bacterial lysate OM-85 (containing 5 mg of standardized lyophilized fractions of *Haemophilus influenzae*, *Streptococcus pneumoniae*, *Klebsiella pneumoniae*, *Klebsiella ozaenae*, *Staphylococcus aureus*, *Streptococcus pyogenes*, *Streptococcus viridans*, and *Neisseria catarrhalis* per capsule), which is used to prevent recurrent respiratory tract infections in children and adults, was shown to reduce the frequency of wheezing attacks in a randomized, double-blind, placebo-controlled, parallel group study encompassing 75 children aged one to six years with a recent history of wheezing attacks (94). Additionally, OM-85 was shown to suppress asthma via a myeloid differentiation primary response 88 protein (MyD88)/TIR-domain-containing adaptor inducing IFN-β (TRIF)-dependent reprogramming of DCs, targeting the epithelium and IL-33-mediated activation of innate like lymphocyte cells type 2 (ILC2s) in the lung (99). However, if OM-85 induces trained immune responses is currently unknown.

Recently, the influence of neonatal Bacillus Calmette-Guérin (BCG) vaccination on asthma development was investigated in the Melbourne Infant Study: BCG for Allergy and Infection Reduction. In this randomized controlled trial, 1272 infants were either vaccinated with BCG or placebo within the first 10 days of life and the incidence of asthma was determined at 5 years of age (95). While in the overall population, neonatal BCG vaccination was associated with a modest reduction in asthma outcomes (14.4% asthmatic children in the BCG group compared to 16.0% in the control group), in high-risk patients the rate of asthma was further decreased in the BCG group compared with the control group (17.6 *vs*. 24.7%) (95). While these results are encouraging, both larger studies and longer observation periods are necessary to evaluate a possible BCG-induced heterologous protection against allergies.

Also, environmental pollutants can induce trained immunity that alters immune responses towards secondary allergen stimulation. Comparing circulating monocytes from healthy and asthmatic children, Movassagh and coauthors recently showed children highly exposed to fine particulate pollutants to have elevated levels of classical (CD14^+^/CD16^-^) monocytes while non-classical circulating monocytes (CD14^lo/-^/CD16^+^) were reduced (100). Immunologically, the fine particulate particles acted as a training stimulus for enriched circulating monocytes that induced enhanced secretion of the pro-inflammatory molecules TNF-α, IL-6, and IL-8 upon secondary stimulation with either HDM or LPS (100). This trained phenotype was controlled epigenetically by enhanced levels of H3K27ac in circulating monocytes (100).

### 3.3 The role of trained immunity in AIT

Since innate immune responses of allergic patients are different from non-allergic controls and environmental factors can change the training status of innate immune cells (as has been described above), this opens the questions (I) whether AIT also influences the trained immune status and (II) whether we can harness inducing trained tolerance or trained anti-inflammatory immune responses for allergy treatment.

It is well established that AIT alters the frequency, phenotype, and function of adaptive immune cells towards restoring immunological tolerance. Namely, AIT dampens allergic symptoms by inducing regulatory T and regulatory B cells which help shifting allergy-related T cell responses away from its Th2-bias [reviewed in (101)]. Additionally, AIT induces allergen-specific IgG_4_-, IgG_2_-, and IgA antibodies and reduces allergen-specific IgE production [reviewed in (101)]. Combined, this leads to the inhibition of IgE-Fc_ϵ_RI crosslinking, reducing degranulation and therefore allergic symptoms [reviewed in (101)].

However, recently also changes in the innate immune system during AIT were described. This metabolic and epigenetic reprogramming in monocytes, APCs, and ILCs was found to be contributing to long-term tolerance (12). Eljaszewicz and colleagues followed innate immune cell frequencies during AIT with either alum-adjuvanted grass- or pollen-AIT products in allergic patients as well as healthy controls (12). They observed an AIT-induced decrease in circulating Th2-promoting ILC2s in allergic patients in comparison to healthy controls (12). Here, allergic patients that underwent AIT had ILC2 levels comparable to healthy controls after completing AIT (12). This was accompanied by an upregulation of Th1-promoting ILC1s, that lasted three years after the start of AIT. They also reported that the first year of AIT reduced frequencies of non-classical (pro-inflammatory) monocytes in favor of intermediate (anti-inflammatory) monocytes which they connected to the observed increase in tolerogenic pDCs and CD141^+^ myeloid DCs (mDCs). Based on these results, in the future, either monitoring the innate immune cell frequencies or identifying specific epigenetic markers in these cells could be used as biomarkers to diagnose allergic diseases or predict long-term treatment responses. Although there were no experiments performed regarding the metabolic state and epigenetic modifications of the cells, the described systemic and long-lasting changes in innate immune cell repertoire suggest that AIT also causes trained tolerance/trained anti-inflammatory immunity.

That training innate immune cells can be beneficial in an allergic setting was also described by Michel et al. (102). The group transferred DCs from mice treated with an antigen-unspecific STAT6-inhibitory peptide (IP) to naïve mice, followed by a ragweed allergic sensitization protocol. In theory, STAT6 inhibition prevents Th2 differentiation (102). Accordingly, naïve mice that received DCs from STAT6 IP-treated animals showed a tolerogenic response that prevented allergic reactions (102). Mechanistically, this suppression of allergic responses was mediated by TGF-β released from the STAT6-IP treated DCs (102). Therefore, these DCs showed a trained tolerogenic phenotype which was non-specific, long-lasting, transferable, and actively inducing regulatory responses during the ragweed sensitization protocol. However, epigenetic modifications were not investigated.

Currently, the adjuvants used in AIT products are limited to aluminum substances and the L-tyrosine-adsorbed TLR4-ligand 3-O-deacylated monophosphoryl lipid A (MPL) [reviewed in (103)]. To our knowledge, so far aluminum-based adjuvants have not been shown to induce pro-tolerogenic trained immunity. Also, in our own previous work alum-adjuvanted AIT products did not induce a metabolic switch in APCs [(104) and Schiller, unpublished data]. On the contrary, Chen et al. have shown that an alum-adjuvanted vaccine trains neonate mouse APCs systemically and long-lastingly, resulting in the induction of Th2-skewed immune response also directed against heterologous antigens, such as OVA (82). In contrast, it is possible that the mixture of L-tyrosine and MPL induces anti-inflammatory trained immune responses. Zimmermann et al. have shown that an L-tyrosine-MPL-adjuvanted AIT product mTOR-dependently activated Warburg metabolism in mouse mDCs (104). This AIT product also induced an mTOR-dependent shift towards Th1-responses in DC:CD4^+^ T cell co-cultures (104). Here, epigenetic modifications have not been investigated.

So far, the focus in AIT was not been on the products’ ability to induce trained immunity but rather the modulation of allergen-specific immune responses in adaptive immune cells. Here, choosing adjuvants that can also induce tolerance via mechanisms of trained immunity might hold potential for AIT.

To improve AIT via innate immune cell modulation, a promising approach may be the use of trained-immunity-based adjuvants to generate so-called trained immunity-based vaccines (TIbVs). In an AIT setting, an allergen would be combined with a tolerance-inducing substance [reviewed in (105)]. Potential adjuvants that have been described to induce trained immune responses in an allergy setting include flagellin, cannabinoids, or mannan. These specifically target and reprogram APCs to induce a tolerogenic response.

One such type of TIbV that is currently investigated in clinical trials are allergoid-mannan conjugates. Benito-Villavilla et al. have shown that these conjugates can reprogram monocytes into tolerogenic DCs (106). Mannan is recognized by the PRRs DC-specific intracellular adhesion molecule-3-grabbing non-integrin (DC-SIGN) and mannose receptor on DCs and internalized together with the allergen. Via metabolic and epigenetic reprogramming, this leads to the induction of allergen-specific Tregs and IgG_2a-_ or IgG_4_ blocking antibodies (106). Treatment with allergoid-mannan conjugates also reprogrammed patient monocytes into tolerogenic APCs (106). Benito-Villavilla et al. described increased glycolysis and OXPHOS, as well as enriched chromatin markers H3K4me3 and H3K27ac near genes of tolerogenic molecules in tolerogenic DCs (106). This example shows how innate immune training via metabolic and epigenetic changes can influence adaptive immune responses and can be beneficial for treatment outcomes.

Angelina et al. have demonstrated that also cannabinoids reprogram human DCs via recognition by cannabinoid receptor 1 (CB1)- and nuclear peroxisome proliferator activated receptor α (PPARα) (107). Cannabinoids promoted tolerogenic DCs that induce FOXP3^+^ Tregs (107). Interestingly, in this process, the mTOR signaling pathway was inhibited and autophagy induced while DC metabolism was shifted towards increased usage of the Krebs cycle and oxidative phosphorylation (107). Here, the cannabinoid WIN55212–2 was shown to prevent peanut sensitization and induce a tolerogenic response in a mouse peanut allergy model (108). Therefore, cannabinoids might also be used as part of TIbVs for AIT, but evidence of training-induced epigenetic modifications was not provided.

In our own pre-clinical research, flagellin, mainly recognized by TLR5 and the NLR family caspase activation and recruitment domain-containing 4 protein (NLRC4) inflammasome, induced tolerogenic responses when fused to either the mugwort allergen Art v 1, the egg white allergen OVA, or the major birch pollen allergen Bet v 1 (109–113). Other groups also used flagellin fused to the cockroach allergen Per a 10 (114). The flagellin fusion protein-trained DCs were able to induce a shift from Th2 to Th1/Treg responses in co-cultures with allergen-specific mouse CD4^+^ T cells (109). This tolerogenic response was shown to be critically dependent on a mTOR-mediated shift in mDC metabolism toward increased rates of glycolysis and the secretion of the immune-suppressing cytokine IL-10 (113, 115). If this tolerogenic response is fueled by epigenetic modifications has not been shown yet. Therefore, the induced metabolic reprogramming of innate immune cells contributes to the potent immune-modulating properties of flagellin as an adjuvant for AIT.

Other approaches to improve AIT via the induction of a tolerogenic phenotype in DCs include the use of either intrinsically tolerogenic nanoparticles (consisting of e.g. poly(lactic-co-glycolic-acid) (PLGA) or carbon-nanotubes) or parasite-derived extracellular vesicles (EVs) (isolated e.g. from *Trichinella* sp*iralis* or *Fasciola hepatica*) [reviewed in (116, 117)]. Depending on their physicochemical properties, nanoparticles can induce a tolerogenic state via promoter hypomethylation and histone modifications [(118) and reviewed in (116, 117)]. In the context of AIT, these approaches have been used to induce tolerance but so far research efforts have mainly focused on investigating either the DC-mediated modulation of Th-responses or epigenetic changes within nanoparticle-treated DCs (119, 120). To this date, the effects of nanoparticles on bone marrow precursors or in a trained immunity setup have not been published [(119, 120) reviewed in (121)]. Parasite-derived EVs are reported to both suppress inflammatory responses in murine DCs and to reduce allergic reactions in mice through the induction of regulatory adaptive immune cells (122, 123). Furthermore, some studies report that parasite-derived excretory/secretory products (including EVs) modulate innate immune cells and their hematopoietic stem cell precursors towards a tolerogenic phenotype, hinting at the potential to induce trained immunity (124, 125). However, likely due to the complexity and heterogeneity of these EVs, other studies have shown a potential to aggravate allergic reactions and first clinical trials so far only showed limited clinical benefits [reviewed in (126)].

All in all, harnessing trained immunity for AIT could improve efficacy and revolutionize allergy treatment. Although multiple *in vitro* studies have been conducted to investigate trained immunity effects of potential adjuvants, and it was recently shown that innate immune cells are modified in patients undergoing AIT, so far there is no comprehensive understanding of the role of trained immunity in AIT.

## 4 Discussion

The discovery of both the existence of and the molecular mechanisms underlying trained immune responses has strongly improved our understanding of how the immune system efficiently prevents infection.

While the highly beneficial contribution of trained immunity to heterologous protection against infectious diseases was repeatedly demonstrated [see the “BCG paradox” for more information (6, 7)], trained immunity may also contribute to pathological processes like chronic inflammatory diseases by inappropriately increasing immune system reactivity (127). Keeping these findings in mind, it is likely that trained immunity programs also contribute to allergic diseases. However, currently the contribution of trained immunity to the establishment, maintenance, and resolution of allergic responses needs more research.

In summary, the currently available literature has clearly and repeatedly shown that the immune system of young allergic or allergy-prone children displays distinct alterations that result in hyperactivity (especially of monocytes) towards exogenous stimuli and may thereby facilitate the subsequent induction of allergen-specific Th2 responses. While certain viral infections and pollutants early in life were shown to predispose towards Th2 responses later in life via mechanisms connected to trained immunity, the data on bacterial infections/vaccines in contrast suggest a trained, allergy-protective effect. However, so far, the available studies are only observatory in nature and did not apply a train-then-challenge design with detailed epigenetic analyses. In future, such studies are needed to conclusively demonstrate the involvement of trained immunity in the observed effects.

Currently, it is not well understood how the principles of trained immunity contribute to treatment success in AIT. Recently, it was demonstrated that AIT induces changes in several innate immune cell types, altering their frequencies and phenotypes towards the levels and phenotypes observed in healthy controls. Also, multiple studies have shown that training innate immune cells can have effects on allergic responses. In future, this beneficial training effect might be harnessed in TIbVs that combine allergens with tolerance inducing substances. Although the potential advantages of these therapeutics have been repeatedly shown *in vitro*, we still lack the understanding of the underlying mechanisms of trained immunity in AIT and how it could be utilized for improving allergy treatment.

Here, additional prospective clinical trials that integrate classical immunological parameters (development of allergen-specific antibody levels, immune status, frequency-, activation status-, and development of cell types) with multiple OMICs approaches (e.g. epigenomics, transcriptomics, metabolomics, proteomics) may shed light on the contribution of trained immunity but also have the tendency to generate huge datasets which are difficult to interpret and extract the essential information from. Therefore, these studies will need to be complemented by mechanistic studies in respective animal models. Together, the obtained results could form the theoretical basis to further improve AIT by not only targeting allergen-specific adaptive immune responses but also modulating underlying Th2-promoting trained immune responses.

## Glossary

2-DG: 2-desoxy glucose
ACL: ATP citrate lyase
AIT: allergen-specific immunotherapy
AKT: protein kinase B
AMPK: AMP (adenosine monophosphate) kinase
APC: antigen-presenting cell
BCG: Bacillus Calmette-Guérin
βFGF: basic fibroblast growth factor
BMDM: bone marrow-derived macrophage
CB1: cannabinoid receptor 1
CCL: CC-chemokine ligand
CD: cluster of differentiation
cDC: conventional DC
c-Myc: c-Myc oncogene
DC: dendritic cell
DC-SIGN: DC-specific intracellular adhesion molecule-3-grabbing non-integrin
EGF: epidermal growth factor
ENO-1: enolase 1
EV: extracellular vesicle
EV-A71: enterovirus A71
Flt1: Fms-related receptor tyrosine kinase 1
GABA: gamma amino butyric acid
GSDMD: gasdermin D
H3K27: histone 3 lysine 27
H3K27ac: H3K27 acetylation
H3K27me3: H3K27 triple methylation
HDM: house dust mite
HIF: hypoxia inducible factor
IDH: iso-citrate dehydrogenase
IFN: interferon
IGF-1: insulin-like growth factor 1
IL: interleukin
ILC: innate-like lymphocyte
IL-4R: IL-4 receptor
IP: inhibitory peptide
IRG1: immune responsive gene 1
iNOS: inducible NO synthetase
JAK: Janus kinase
JMJD3: Jumonji domain-containing protein-3
KDM6B: lysine demethylase 6B
LPS: lipopolysaccharide
M2: alternatively activated
MBP-1: myc promoter-binding protein 1
mDC: myeloid DC
MHC: major histocompatibility complex
moDC: monocyte-derived DC
MPL: 3-O-deacylated monophosphoryl lipid A
MTA: 5’-deoxy-5’-(methylthio) adenosine
mTOR: mammalian target of rapamycin
mTORC2: mTOR complex 2
MuHV-4: murid herpesvirus 4
MyD88: myeloid differentiation primary response 88 protein
NLR: nucleotide binding leucine-rich repeat-like receptor
NLRC4: NLR family caspase activation and recruitment domain-containing 4 protein
NLRP3: NLR family pyrin domain containing 3 protein
NO: nitric oxide
Nrf2: nuclear factor erythroid 2-related factor 2
OVA: ovalbumin
PAMPs: pathogen associated molecular patterns
PBMC: peripheral blood mononuclear cell
pDC: plasmacytoid dendritic cell
PDGF: platelet-derived growth factor
PGE2: prostaglandin E2
PIGF: placental growth factor
PHA: phytohemagglutinin
PHD: prolyl hydroxylase domain
PI3K: phosphatidylinositol 3-kinase
PLGA: poly(lactic-co-glycolic-acid)
PPAR(α): nuclear peroxisome proliferator activated receptor (α)
PRR: pattern recognition receptors
PVM: pneumonia virus of mice
RLR: RIG-I-like receptor
ROS/NOS: reactive oxygen/nitrogen species
RSV: respiratory syncytial virus
STAT: signal transducer and activator of transcription
TET: ten-eleven translocation
TGF-β: transforming growth factor beta
TIbVs: trained immunity-based vaccines
TLR(4): “Toll”-like receptor (4)
TNF: tumor necrosis factor
TRIF: TIR (Toll/IL-1 receptor)-domain-containing adaptor inducing IFN-β
TSLP: induced thymic stromal lymphopoietin
VEGF-A: vascular endothelial growth factor A.
